# Emerging roles of histone deacetylases in adaptive thermogenesis

**DOI:** 10.3389/fendo.2023.1124408

**Published:** 2023-02-16

**Authors:** Ruonan Zhou, Yue Cao, Yingying Xiang, Penghua Fang, Wenbin Shang

**Affiliations:** ^1^ Department of Endocrinology, Jiangsu Province Hospital of Chinese Medicine, The Affiliated Hospital of Nanjing University of Chinese Medicine, Nanjing, China; ^2^ Key Laboratory for Metabolic Diseases in Chinese Medicine, First College of Clinical Medicine, Nanjing University of Chinese Medicine, Nanjing, China

**Keywords:** histone deacetylase, obesity, adaptive thermogenesis, adipose tissue, diabetes

## Abstract

Brown and beige adipose tissues regulate body energy expenditure through adaptive thermogenesis, which converts energy into heat by oxidative phosphorylation uncoupling. Although promoting adaptive thermogenesis has been demonstrated to be a prospective strategy for obesity control, there are few methods for increasing adipose tissue thermogenesis in a safe and effective way. Histone deacetylase (HDAC) is a category of epigenetic modifying enzymes that catalyzes deacetylation on both histone and non-histone proteins. Recent studies illustrated that HDACs play an important role in adipose tissue thermogenesis through modulating gene transcription and chromatin structure as well as cellular signals transduction in both deacetylation dependent or independent manners. Given that different classes and subtypes of HDACs show diversity in the mechanisms of adaptive thermogenesis regulation, we systematically summarized the effects of different HDACs on adaptive thermogenesis and their underlying mechanisms in this review. We also emphasized the differences among HDACs in thermogenesis regulation, which will help to find new efficient anti-obesity drugs targeting specific HDAC subtypes.

## 1 Introduction

Obesity and related metabolic disorders are established risk factors for multiple non-communicable diseases (NCDs) such as type 2 diabetes mellitus, fatty liver disease, hypertension and stroke, thereby impairing patients’ quality of life and life expectancy and creating a great economic burden on society (1–3). A disturbance of energy homeostasis is the primary cause of obesity. When energy intake exceeds usage, obesity and its comorbidities develop. However, conventional interventions on calorie intake are poorly effective and easily promote weight regain due to the intricate and persistent metabolic adaptations defending against weight loss in the body (3–5). Hence, alternative strategies based on increasing energy expenditure to remodify metabolic efficiency in key metabolic organs are urgently needed.

Adipose tissue plays a pivotal role in the development of obesity (6, 7). There are two general types of adipose tissue in mammals: white adipose tissue (WAT) and brown adipose tissue (BAT). Each is composed of functionally and morphologically distinct adipocyte populations. While the main function of WAT is to store energy as triglycerides in unilocular white adipocytes, brown adipocytes within BAT characterized by multilocular lipid droplets and an abundance of mitochondria dissipate energy in the form of heat through adaptive thermogenesis (8). Adaptive thermogenesis is primarily supported by uncoupling protein 1 (UCP1), which is uniquely expressed within the inner membrane of mitochondria in brown adipocytes. UCP1 is a dimer composed of two 32 kDa subunits that forms a proton leak to uncouple oxidative phosphorylation and generates heat as a by-product (9–11).

**Figure 1:**
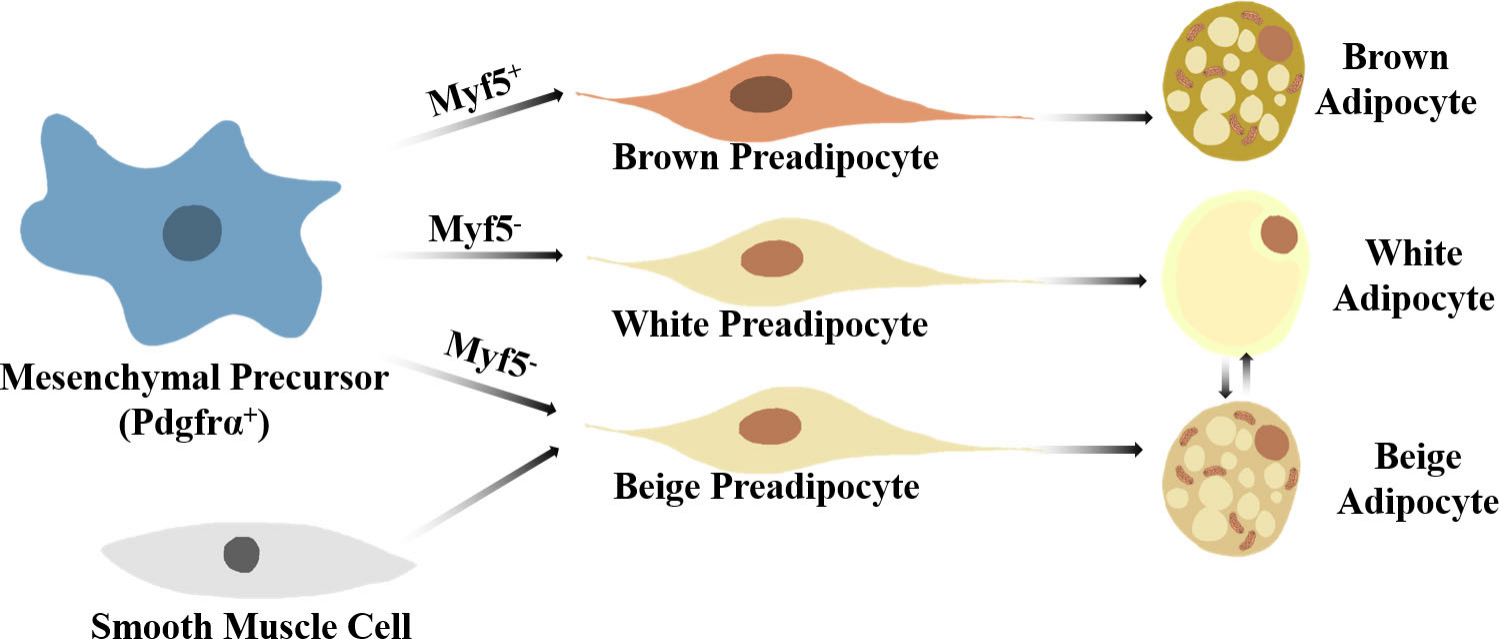
A brief view on thermogenic adipocyte differentiation. Brown adipocytes arise from Myf5^+^Pdgfrα^+^ mesenchymal precursors, while white and beige preadipocytes develop from Myf5^-^Pdgfrα^+^ mesenchymal precursors. Specific stimuli can induce beige-white adipocyte phenotype switch in WAT. Smooth muscle-like precursor cells can also give arise to beige adipocytes.

Specific stimuli, for instance, cold exposure, not only promote thermogenic capacity as well as the differentiation of brown adipocytes but also induce a kind of brown-like adipocyte in WAT named beige adipocytes (12, 13). Although beige and brown adipocytes have similar thermogenic capacity and morphological features, the two types of adipocytes have significantly different localization and origin (**Figure 1**) (8, 14, 15).

As the major forms of adaptive thermogenesis, brown adipose tissue thermogenesis and white adipose beigeing are proved effective to defense against metabolism disorder induced by obesity in both human and rodents. Results of positron emission tomography/computed tomography examinations confirmed that fluorodeoxyglucose-detected brown adipose tissue content is negatively associated with age, BMI and diabetes status in adult volunteers (16). In diet induced obesity (DIO) mouse models, activation of BAT by cold exposure or β3-adrenoceptor activator also increased the insulin sensitivity of peripheral tissues and reduced ectopic lipid deposition in the liver (17, 18). On the other hand, the beigeing of WAT regulates energy homeostasis not merely through promoting thermogenesis, but also by ameliorating obesity induced fibrosis, hypoxia and insulin resistance in adipose tissue (19). Furthermore, beige adipocytes have a distinct developmental lineage from brown adipocytes, ensuring WAT beigeing a broader application prospect in obesity treatment in populations with low BAT content, particularly the elderly (20).

In view of the rising obesity epidemic, mechanisms regulating adaptive thermogenesis are of significant importance for the treatment of obesity and related metabolism disorders (21). Nevertheless, common adipose thermogenesis promoters involving sympathetic nervous system activation like thyroxin and noradrenaline lack tissue specificity, and their side effect of multi-system sympathetic excitement makes the risks far outweigh the benefits (22, 23). Extensive exploration of safe and effective agents promoting adipose tissue thermogenesis is still eagerly required.

Recent studies have shown the critical role of histone deacetylases (HDACs) in obesity pathology. HDACs are responsible for lysine residue deacetylation of a wide range of both histone and non-histone proteins, and have been involved in various biological processes, including adaptive thermogenesis (24). In this review, we discuss both transcriptional and non-transcriptional regulation of adaptive thermogenesis by HDACs along with their underlying mechanisms, with specific emphasis on the disparate roles among HDAC subtypes. We also highlight the effects of HDAC inhibitors on adaptive thermogenesis.

## 2 Overview of histone deacetylases

It has long been recognized that histone acetylation neutralizes the positively charged lysine residues of the histone N termini, which could induce nucleosomes to unfold, thus opening the transcription of specific genes and enhancing their expression level (25). Since Taunton et al. named the first histone deacetylase HDAC1 in 1996, a total of 18 different HDACs categorized into 4 classes have been identified in mammals so far (**Table 1**) (26).

**Table 1:** Overview of existing 18 different HDACs.

Class	Subtype	Subcellular Location	Structural Feature
I	HDAC1	Nucleus	Zn^2+^-dependent
	HDAC2	Nucleus and cytoplasm	Zn^2+^-dependent
	HDAC3	Nucleus and cytoplasm	Zn^2+^-dependent
	HDAC8	Nucleus and cytoplasm	Zn^2+^-dependent
IIa	HDAC4	Nucleus and cytoplasm	Zn^2+^-dependent
	HDAC5	Nucleus and cytoplasm	Zn^2+^-dependent
	HDAC7	Nucleus and cytoplasm	Zn^2+^-dependent
	HDAC9	Nucleus	Zn^2+^-dependent
IIb	HDAC6	Cytoplasm and nucleus	Zn^2+^-dependent
	HDAC10	Cytoplasm and nucleus	Zn^2+^-dependent
III	SIRT1	Nucleus and cytoplasm	NAD^+^-dependent
	SIRT2	Cytoplasm and nucleus	NAD^+^-dependent
	SIRT3	Mitochondrion matrix and cytoplasm	NAD^+^-dependent
	SIRT4	Mitochondrion matrix	NAD^+^-dependent
	SIRT5	Mitochondrion, cytoplasm and nucleus	NAD^+^-dependent
	SIRT6	Nucleus and endoplasmic reticulum	NAD^+^-dependent
	SIRT7	Nucleus and cytoplasm	NAD^+^-dependent
IV	HDAC11	Nucleus	Zn^2+^-dependent

To play their role in histone deacetylation, HDACs are required to be located in the nucleus, where their predominant substrate can be found. Many HDACs, however, have been found to be expressed in cellular components other than the nucleus. Class I HDACs (HDAC-1, -2, -3, -8) and Class IV HDACs (HDAC11) mainly express in the nucleus, and class IIb HDACs (–6, –10) distribute in the cytoplasm. Class IIa HDACs (–4, –5, –7, –9) can shuttle between the nucleus and cytoplasm, while class III HDACs, namely NAD^+^ dependent Sirtuin deacetylase (SIRT1-7) spread in different cell components (27).

It is demonstrated that the nuclear translocalization of HDACs could be induced through a nuclear localization signal (NLS) or by colocalization with other proteins. However, some HDACs lack NLS and could only be located in the cytoplasm (28). Further research uncovered that lysine acetylation is a widespread protein post-translational modification happening on both histone and non-histone proteins (24). Indeed, mass spectrometry-based studies proved that HDACs are also responsible for non-histone protein deacetylation, which involves various cellular processes relevant to physiology and disease, such as cell division, signal transduction and metabolism (24, 29). Therefore, more and more efforts have been made to explain the relationship between HDACs and different biological processes and the underlying mechanisms so as to seek valuable therapeutic strategies for tumors, metabolic diseases, cardiovascular diseases, et al. (30–33).

## 3 Histone deacetylases in adaptive thermogenesis

After Gao et al. provided sodium butyrate (a pan-HDAC inhibitor) as dietary supplementation to DIO mice and first reported the anti-insulin resistance along with the BAT thermogenesis promoting effects of sodium butyrate in 2009, an increasing number of explorations have been made to illustrate the regulatory effects of HDACs on adaptive thermogenesis and their underlying mechanisms (34–36). Available evidence supports the notion that HDACs are capable of affecting both brown adipose tissue thermogenesis and white adipose tissue beigeing. The mechanisms of HDACs regulating adaptive thermogenesis involve transcriptional as well as non-transcriptional control.

### 3.1 Direct transcriptional control of adaptive thermogenesis

#### 3.1.1 Regulation on transcription factors

Transcription factors are significant participants in the process of transcription initiation. Transcriptional factors involving in biological processes including adipogenesis, lipolysis, oxidative phosphorylation (OXPHOS) and et al. coregulate adipose tissue thermogenesis. To date, HDACs have been shown to affect adipose tissue thermogenesis by adjusting two main transcription factors–PRDM16 [PRDI-BFI (positive regulatory domain I-binding factor 1) and RIZ1 (retinoblastoma protein-interacting zinc finger gene 1) homologous domain containing protein 16] and PGC-1α (Peroxisome proliferators-activated receptor γ coactivator 1α) through acetylation and non-acetylation ways.

In mouse primary brown adipocytes, immunoprecipitation assays demonstrated that HDAC3 directly interacts with PRDM16, a key protein regulating adipose differentiation (37, 38). PRDM16 promotes adaptive thermogenesis gene expression through interactions not only with various related transcription factors such as PPARγ, PGC-1α, ZFP516 et al., but also with several small noncoding RNAs (miRNAs) participating in adipocyte differentiation (38). The treatment of HDAC3-selective inhibitor RGFP966 increased the expression of thermogenic proteins such as UCP1 in primary mouse brown adipocytes, and this induction was blunted in adipocytes isolated from *Prdm16* knockout mice, which suggests that the regulation of thermogenesis in brown adipocytes by HDAC3 is closely related to the function of PRDM16 (37). In addition, SIRT1 is also found to induce the deacetylation of Lys268 and 293 sites of PPARγ, which recruits more PRDM16 binding to PPARγ and promotes white adipose beigeing (39).

PGC-1α is one of the major transcription factors regulating mitochondrial biogenesis along with oxidative phosphorylation, thus playing an important role in brown and beige adipogenesis (40). In mouse brown adipose tissue, HDAC3 activates PGC-1α through both deacetylation and upregulation protein expression to coactivate ERRα (Oestrogen-related receptor α), which triggers the transcriptional loop of *Ucp1*, and other OXPHOS genes (41). It has also been shown that in pre-brown adipocytes isolated from mice, SIRT6 could interact with phosphorylated activating transcription factor 2 (ATF2) and induce p-ATF2 binding to the *Pgc-1α* promoter, thus enhancing PGC-1α expression. Adipose-specific *Sirt6* knockout mice developed an obesity, insulin resistance and severe fatty liver phenotype due to impaired white and brown adipose tissue adaptive thermogenesis (42). In HIB-1B pre-brown adipocytes, overexpression of *Sirt3*, another member of the Sirtuin family, has also been shown to promote the protein expression of PGC-1α and UCP1 (43). Conversely, overexpression of *Pgc-1α* in HIB-1B pre-adipocytes similarly upregulated the protein levels of SIRT3 and UCP1. However, overexpression of *Pgc-1α* in *Sirt3* silenced mouse embryonic fibroblasts but failed to induce the expression of thermogenic genes such as *Ucp1* (44). The evidence above indicates the fact that SIRT3-PGC-1α interaction is integral for PGC-1α to regulate the browning process, which is critical for brown adipose tissue thermogenesis.

#### 3.1.2 Regulation on chromatin remodeling

Chromatin remodeling is an important component of pre-transcriptional regulation. There are two common forms of chromatin remodeling: covalent modification of histones and ATP-dependent chromatin remodeling. Ample evidence supports the idea that HDACs play essential roles in chromatin remodeling and thereby affect adaptive thermogenesis.

Histone covalent modification usually occurs at terminal residues and includes the forms of methylation, acetylation, phosphorylation and ubiquitination (45). HDACs perform the inhibitory effect on transcription process mainly through deacetylating ϵ-amino on the side-chain of lysine residues in histones (27). Data from Ferrari et al. showed that adipose-specific knockout of *Hdac3* promotes the production of multilocular beige adipocytes in the inguinal white adipose tissue of C57/BL6 mice, and this process was induced by the increase of histone 3 lysine 27 (H3K27) acetylation in the *Pparγ* enhancer region. Moreover, acetyl coenzyme A required for H3K27 acetylation could be provided by boosted cleavage of citrate and fatty acid β-oxidation triggered by *Hdac3* silence (46). Similar to HDAC3, HDAC1 is another HDAC from Class I that has an inhibitory effect on adaptive thermogenesis. In a 7-day cold exposure test on A/J mice, researchers found blunted *Hdac1* gene expression in contrast to increasing *Ucp1* gene expression in interscapular brown adipose tissue. Further study demonstrated that in mice brown adipose tissue treated with β3-adrenergic agonist, HDAC1 dissociates from promoter regions of *Ucp1* and *Pgc-1α*, and at the same time recruits lysine-specific demethylase 6A/ubiquitously transcribed tetratricopeptide repeat on chromosome X (KDM6A/UTX) to these promoter regions, causing a high level of H3K27 acetylation along with an impaired level of H3K27 trimethylation, which activates the transcription of *Ucp1* and *Pgc-1α*, and subsequently promoting brown adipose tissue thermogenesis (47). Moreover, the Sirtuin family member SIRT3 has recently been shown to be an inhibitor of mitochondrial calcium overload *via* a mechanism of lowering the H3K27ac level on the mitochondrial calcium uniporter (MCU) promoter in an AMPK-dependent manner, thereby preventing obesity or age-related whitening of brown adipose tissue (48).

The chromatin remodeling complex is a vital tool for the physical modification of chromatin. Chromatin remodeling complexes alter the binding state of histones to DNA in an ATP-dependent manner by interacting with chromatin, making DNA-binding proteins more accessible to nucleosomal DNA for transcription. In this process, enzymes responsible for histone modification usually work together with chromatin remodeling complexes (49). BRD2, a member of the BET family, has been shown to process chromatin regulatory activity similar to that of the chromatin remodeling complex (50). Meanwhile, by electively recognizing acetyl lysine, BRD2 could significantly suppress brown adipocyte differentiation. HDAC11 was observed to directly interact with the ET region of the BRD2 protein. In pre-HIB1B brown adipocytes, overexpression of *Hdac11* impaired the acetylation of *Ucp1* enhancer H3K27, thus inhibiting the gene transcription of *Ucp1*. Silencing *Brd2* blunted the inhibitory effect of *Hdac11* overexpression on thermogenesis in HIB1B brown adipocytes and, in the meantime, increased the expression of adipose differentiation-related proteins PPARγ and C/EBPα and promoted brown adipocyte differentiation, which suggested that HDAC11 may inhibit the thermogenic effect of adipose tissue through BRD2 (51).

### 3.2 Other regulation of adaptive thermogenesis

Recently, studies have begun to focus on the role of HDACs in areas other than histone deacetylation and transcriptional regulation. Certain evidence suggests that HADCs regulate cell signaling transduction by regulating multiple proteins involved in adipocyte function, thereby influencing adaptive thermogenesis.

In adipocytes, signals of β3-adrenergic receptor (β3-AR) activation could be transduced by Gs proteins and the cyclic adenosine monophosphate (cAMP)-protein kinase A (PKA) pathway, hence promoting the expression of UCP1 for thermogenesis (52). It was shown that *Hdac6* knockdown significantly downregulated the expression of cAMP and UCP1 in mouse brown adipose tissue and impaired brown adipose tissue thermogenesis. Treating primary brown adipocytes isolated from *Hdac6* knockout mice and cultured *in vitro* with forskolin (an activator of cAMP) rescued the expression of UCP1, which suggests that the effects of HDAC6 on adaptive thermogenesis are closely associated with the cAMP-PKA pathway (53).

Other than acetyl, many HDACs exhibit substrate specificity for other acyl groups. Gravin-α/A kinase–anchoring protein 12 is a kind of cytoplasmic scaffolding protein. Lysine myristoylation of gravin-α drives β-ARs to lipid raft membrane microdomains, which triggers PKA pathway activation and downstream thermogenic gene expression (54). Gravin-α was recently defined as the second HDAC11 substrate to demyristoylation, and HDAC11-mediated gravin-α demyristoylation was found in both murine and human adipose tissue, indicating a role for HDAC11 in adipocyte adrenergic signal transduction independent of deacetylation activity (55).

Fibroblast growth factor 21 (FGF21), a member of the fibroblast growth factor family, is a hormone secreted by multiple tissues, including liver and adipose tissue. FGF21 has the function of promoting fatty acid oxidation and enhancing adaptive thermogenesis (56, 57). *Hdac9* knockout mice showed increasing FGF21 expression in subcutaneous white adipose tissue as well as a higher percentage of beige adipocytes and an ascending energy output (58). However, there is also evidence suggesting that the effect of HDAC9 on adipocyte differentiation may not be dependent on the deacetylation activity of HDAC9 (59). Further studies are required to determine the mechanism by which HDAC9 promotes FGF21 expression and white adipose tissue beigeing.

Aging is another important contributor to impaired adipose tissue thermogenesis and the development of obesity. The process of aging involves various signaling pathways, especially the p53/p21 pathway (60). Vuong et al. isolated adipose tissue-derived mesenchymal stem cells (AT-MSCs) from infants and older volunteers (70 to 80 years old) and attempted to differentiate them into beige adipocytes *in vitro* using differentiation media. The results showed that AT-MSCs from elder volunteers exhibited an obviously weaker ability to be induced into beige adipocytes as well as lower expression of the SIRT1 protein than the infant group. In contrast, overexpression of the Sirt1 gene in AT-MSCs could improve the ability of AT-MSCs from elder volunteers to differentiate into beige adipocytes and, at the same time, down-regulate p53/p21 pathway-related protein expression, which indicates that the impaired ability of white adipose tissue to beige in the elderly is closely related to SIRT1 and its regulation of the p53/p21 pathway (61).

Chronic inflammation in adipose tissue caused by obesity is widely regarded as a major contributor to impaired adaptive thermogenesis (62). Moderate *Sirt1* overexpression rescued insulin sensitivity as well as thermogenic-related responses in brown adipose tissue in C57/BL6 mice injected with a low dose of bacterial lipopolysaccharide (LPS) to mimic endotoxemia. *In vitro*, *Sirt1* overexpression in primary murine brown adipocytes exposed to a macrophage-derived pro-inflammatory conditioned medium consistently promoted mitochondrial respiration, fatty acid oxidation, and norepinephrine-mediated UCP-1 expression, indicating that targeting SIRT1 may have a therapeutic value in the treatment of inflammation-induced thermogenesis deficiency (63).

### 3.3 The multifaceted role of HDACs in adaptive thermogenesis

**Figure 2:**
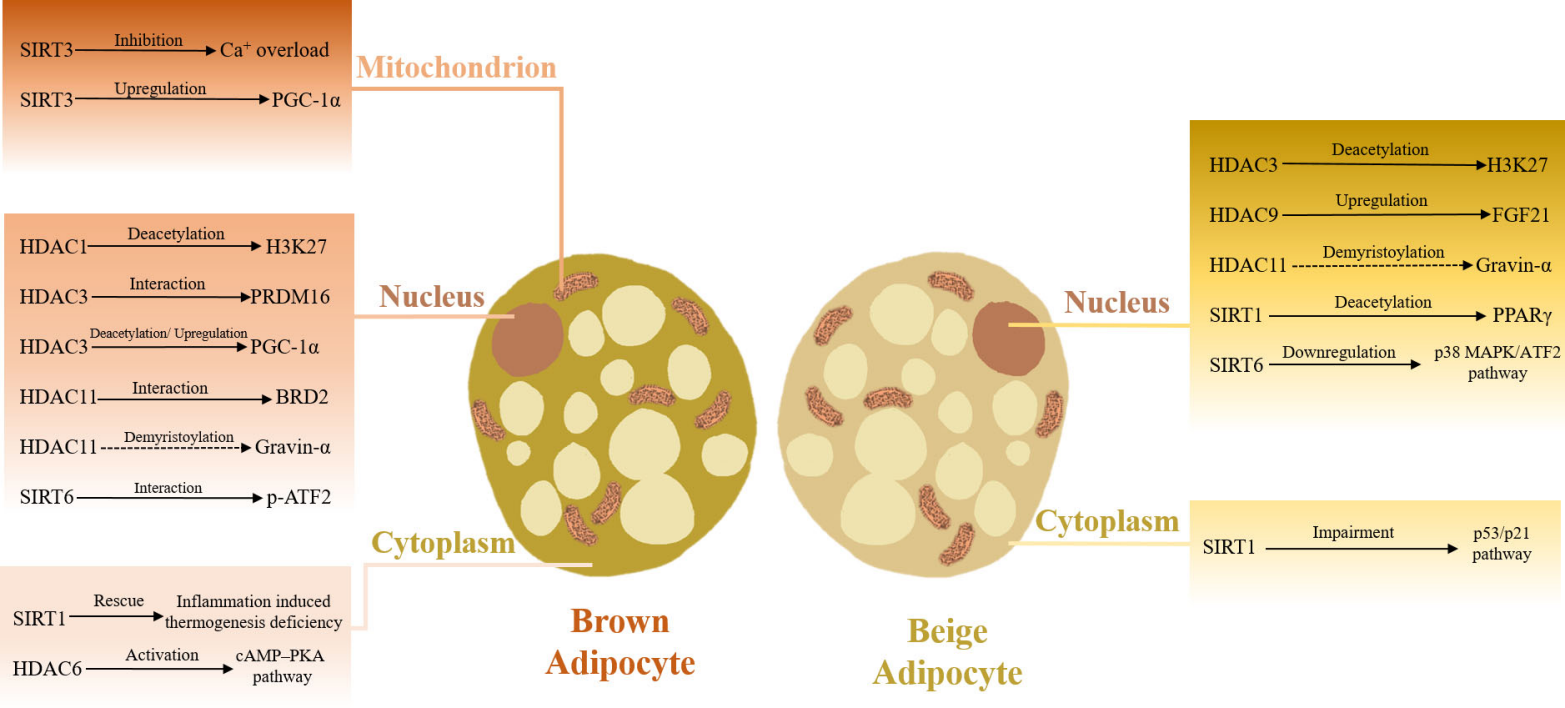
HDACs in adaptive thermogenesis. HDACs located in various cellular components orchestrate adaptive thermogenesis.

As noted above, different classes and subtypes of HDACs show certain complexity and diversity in their effects and underlying mechanisms of adipose tissue thermogenesis regulation (**Table 2**; **Figure 2**).

**Table 2:** HDACs in adaptive thermogenesis.

HDAC Subtype	Key Findings	Citation
HDAC1	HDAC1 dissociates from UCP1 and PGC-1α promoters in brown thermogenic program, leading to increased H3K27 acetylation and decreased H3K27me3 levels of these promoters.	(47)
HDAC3	HDAC3 suppresses white adipose tissue beigeing process by deacetylating H3K27 on PPARγ and UCP1 enhancers.	(46)
	HDAC3 suppresses brown adipocyte thermogenesis by interacting with PRDM16 directly.	(37)
	HDAC3 activates PGC-1α through both deacetylation and upregulation protein expression to coactivate ERRα, which help maintain the capacity for thermogenesis in brown adipose.	(41)
HDAC6	HDAC6 promotes brown adipose tissue thermogenesis through cAMP–PKA pathway.	(53)
HDAC9	HDAC9 gene deletion prevents HFD induced FGF21 downregulation and promotes subcutaneous adipose beigeing independent of its deacetylase enzymatic activity.	(58, 59)
HDAC11	HDAC11 suppresses expression of UCP1 and PGC-1α in brown adipocytes and negatively regulates thermogenesis by physical association with BRD2.	(51)
	HDAC11-mediated gravin-α demyristoylation drives β-ARs to lipid raft membrane microdomains, thus triggering PKA pathway activation and downstream thermogenic gene expression.	(55)
SIRT1	SIRT1 promotes white adipose beigeing by deacetylating PPARγ on Lys268 and Lys293 and recruiting PRDM16 to PPARγ.	(39)
	SIRT1 overexpression restored the beige differentiation ability by impairing p53/p21 pathway in adipose tissue mesenchymal stem cells from elderly donors.	(61)
	Moderate SIRT1 overexpression rescued inflammation induced thermogenesis deficiency in both primary brown adipocytes and mice brown adipose tissue.	(63)
SIRT3	SIRT3 promotes brown adipose tissue thermogenesis by elevating PGC-1α and UCP-1 expression.	(43)
	SIRT3 expression is controlled by PGC-1α and this action is an essential component of PGC-1α induced brown adipocyte differentiation.	(44)
	SIRT3 inhibites mitochondrial calcium overload through reducing H3K27ac level on the *Mcu* promoter in an AMPK-dependent manner, thus preventing obesity or age related whitening of brown adipose tissue	(48)
SIRT6	SIRT6 promotes brown adipose tissue thermogenesis and white adipose tissue beigeing by recruiting and interacting with phospho-ATF2 to activate PGC-1α expression.	(42)
	SIRT6 absence in adipocytes impairs the thermogenesis ability as well as the p38 MAPK/ATF2 signaling of adipose tissue under intermittent fasting.	(64)

Class I and IV HDACs are widely recognized as adaptive thermogenesis molecular breaks. In research performed by Galmozzi et al., 2-week treatment with the pan-class I HDAC inhibitor MS-275 promoted white adipose tissue beigeing and improved oxygen consumption as well as energy output, thus ameliorating insulin resistance and ectopic fat deposition in the liver of db/db mice (65). Similarly, in diet-induced obesity (DIO) mice, they further validated that MS-275 treatment decreased the body weight and white adipose tissue volume, better maintained the core body temperature during cold exposure and significantly increased the expression of UCP1 in both white and brown adipose tissue (66). Also, the capacity of MS-275 to modulate H3K27 acetylation on enhancer regions regulating *Pparγ* and *Ucp1* genes was confirmed in C3H/10T1/2 adipocytes differentiated *in vitro* (67). The only member of class IV HDAC is HDAC11. In DIO mice, HDAC11 absence was also shown to promote brown adipose tissue thermogenesis and white adipose tissue beigeing and ameliorate high-fat-induced obesity. Unlike other classes of HDACs, the deacetylation activity of class IV HDAC is relatively weak, and as such, HDAC11 inhibitors might have a greater prospective in obesity treatment because of their relatively fewer side effects (51, 55).

Different from class I and IV HDACs, class III HDACs (the Sirtuin family) are usually promoters of adaptive thermogenesis. SIRT1, 3, and 6 have been proven to be capable of increasing brown adipose tissue thermogenesis and improving insulin resistance in DIO mice (42–44, 68). While SIRT1 and 6 could also improve obesity-related metabolic disorders in DIO mice by promoting white adipose tissue beigeing (39, 42, 64).

However, the effect of class II HDACs on adipose tissue thermogenesis is not uniform. Though treatment with the pan-class II HDACs inhibitor MC-1568 reduced the expression of the Ucp1 gene in pre-HIB-1B brown adipocytes, existing evidence shows that the regulation of adaptive thermogenesis by class II HDACs varies according to different subtypes (69). As previously mentioned, *Hdac6* knockdown significantly downregulated cAMP and UCP1 expression in mouse brown adipose tissue, whereas *Hdac9* absence promoted subcutaneous adipose tissue UCP1 expression in DIO mice by increasing FGF21 levels (53, 58). Fenfen et al. silenced *Hdac1* to *Hdac11* using siRNA in HIB-1B pre-brown adipocytes, and the results showed that *Hdac6*, *7*, and *10* knockdown caused a slight decrease in *Ucp1* gene expression, while *Hdac5* and *9* knockdown upregulated *Ucp1* gene expression, which might be explained by these HDACs possessing different target proteins and modification positions (47). There is also another hypothesis that class II HDACs (HDAC4-7, 9) are a class of pseudo-enzymes, for class II HDACs only have weak or even no deacetylation activity of their own and rely mainly on the recruitment of class I HDACs to complete the deacetylation reaction (70). Hence, the regulation of adipose tissue thermogenesis by class II HDACs may be dependent on their non-deacetylation functions (59). But no available evidence could confirm this idea directly.

## 4 Conclusion and perspectives

In recent years, the roles of HDACs in regulating adipose tissue thermogenesis and their underlying mechanisms have made remarkable progress and are causing increasing concern. However, there are still some issues that require further exploration. Firstly, most of the studies are at *in vitro* or animal levels, and the effect of HDACs on the regulation of adipose tissue thermogenesis is not yet supported by sufficient clinical trials. Although several clinical studies have been conducted on the application of HADC-based drugs in metabolic diseases such as obesity, there is still a long way to go from lab research to clinical use (71).

Secondly, although evidence suggests that several HDAC-based drugs, such as the HDAC inhibitor sodium phenylbutyrate, SAHA and the SIRT agonist resveratrol can significantly improve adipose tissue distribution and peripheral tissue insulin sensitivity in obesity patients, whether these therapeutic effects are thermogenesis-related remains to be further investigated (72–74).

Furthermore, the regulation of adipose thermogenesis differs among different classes of HDACs, and even different subtypes of HDACs in the same class have distinct effects on adaptive thermogenesis. Therefore, specific HDAC-targeting drugs based on subtypes can not only attenuate or eliminate the possible adverse effects caused by pan-HDAC inhibition, such as thrombocytopenia, nausea, and fatigue, but also promote adaptive thermogenesis effectively. Judging from this, searching for safe and efficient drugs targeting different HDAC subtypes to modulate adaptive thermogenesis will remain a key issue to be explored in the future.

In conclusion, targeting HDACs has a great potential to be an effective therapeutic strategy for obesity and related metabolic disorders. However, further in-depth studies are still eagerly required to fully elucidate the role of HDACs in adaptive thermogenesis.

## Author contributions

RZ interpreted the data and wrote a draft of this manuscript. YC and YX assisted in the interpretation of the data. WS and PF contributed to the design of the study and revision of this manuscript. All authors contributed to the article and approved the submitted version.
